# Phytochemical analysis and protective effects of *Vaccinium macrocarpon* (cranberry) in rats (*Rattus norvegicus*) following ethylene oxide-induced oxidative insult

**DOI:** 10.1080/21655979.2021.1955528

**Published:** 2021-08-04

**Authors:** Mahmood Rasool, Arif Malik, Muhammad Abdul Basit Ashraf, Rabia Mubbin, Ujala Ayyaz, Sulayman Waquar, Muhammad Asif, Muhammad Umar, Gan Siew Hua, Zafar Iqbal, Hina Alam, Niaz M. Achakzai

**Affiliations:** a Center of Excellence in Genomic Medicine Research, Faculty of Applied Medical Sciences, King Abdulaziz University, Jeddah, Saudi Arabia; b Department of Medical Laboratory Technology, Faculty of Applied Medical Sciences, King Abdulaziz University, Jeddah, Saudi Arabia; c Institute of Molecular Biology and Biotechnology (IMBB), University of Lahore, Lahore, Pakistan; d Department of Biotechnology and ORIC, BUITEMS, Quetta, Pakistan; e Department of Livestock & Dairy Development, Veterinary Research Institute, Quetta, Pakistan; f School of Pharmacy, Monash University Malaysia, Bandar Sunway, Selangor Darul Ehsan, Malaysia; g Clinical Laboratory Sciences Program, College of Applied Medical Sciences, King Saud Bin Abdulaziz University for Health Sciences/KAIMRC/SSBMT, National Guard Health Affairs, King Abdulaziz Medical City, Al-Ahsa, Saudi Arabia; h Pakistan Institute of Medical Sciences, Islamabad Pakistan; i Forensic Medicine Directorate (FMD), Ministry of Public Health, Kabul, Afghanistan; j Department of Molecular Biology, City Medical Laboratory, Kabul, Afghanistan

**Keywords:** Ethylene oxide, superoxide oxide dismutase, catalase, glutathione, advanced oxidative protein products, malondialdehyde

## Abstract

The *Vaccinium* genus comprises more than 126 genera of perennial flowering plants that are commonly adapted to poor and acidic soils or epiphytic environments. Their molecular and genomic characterization is a result of the recent advent in next-generation sequencing technology. In the current research, extracts were prepared in different media, such as petroleum ether, methanol and ethanol. An extract of *Vaccinium macrocarpon* (cranberry) was used at a dose of 200–400 mg/kg by weight (B.wt). Levels of oxidative stress markers, i.e., superoxide dismutase (SOD), catalase (CAT), glutathione (GSH), advanced oxidation protein products (AOPPs) and malondialdehyde (MDA), were measured. A histopathological study of six vital organs in rats was also conducted. The results indicated that the antioxidant levels were lower in the group given only ethylene oxide (EtO) but higher in the groups receiving cranberry extract as a treatment. Major improvements were also observed in stress markers such as advanced oxidation protein products (AOPPs) and MDA following cranberry treatment. Histopathological changes induced by EtO were observed in the heart, kidney, liver, lung, stomach and testis and were reversed following cranberry treatment. The major toxic effects of EtO were oxidative stress and organ degeneration, as observed from various stress markers and histopathological changes. Our study showed that this extract contains strong antioxidant properties, which may contribute to the amelioration of the observed toxic effects.

## GRAPHICAL ABSTRACT

**Figure:**
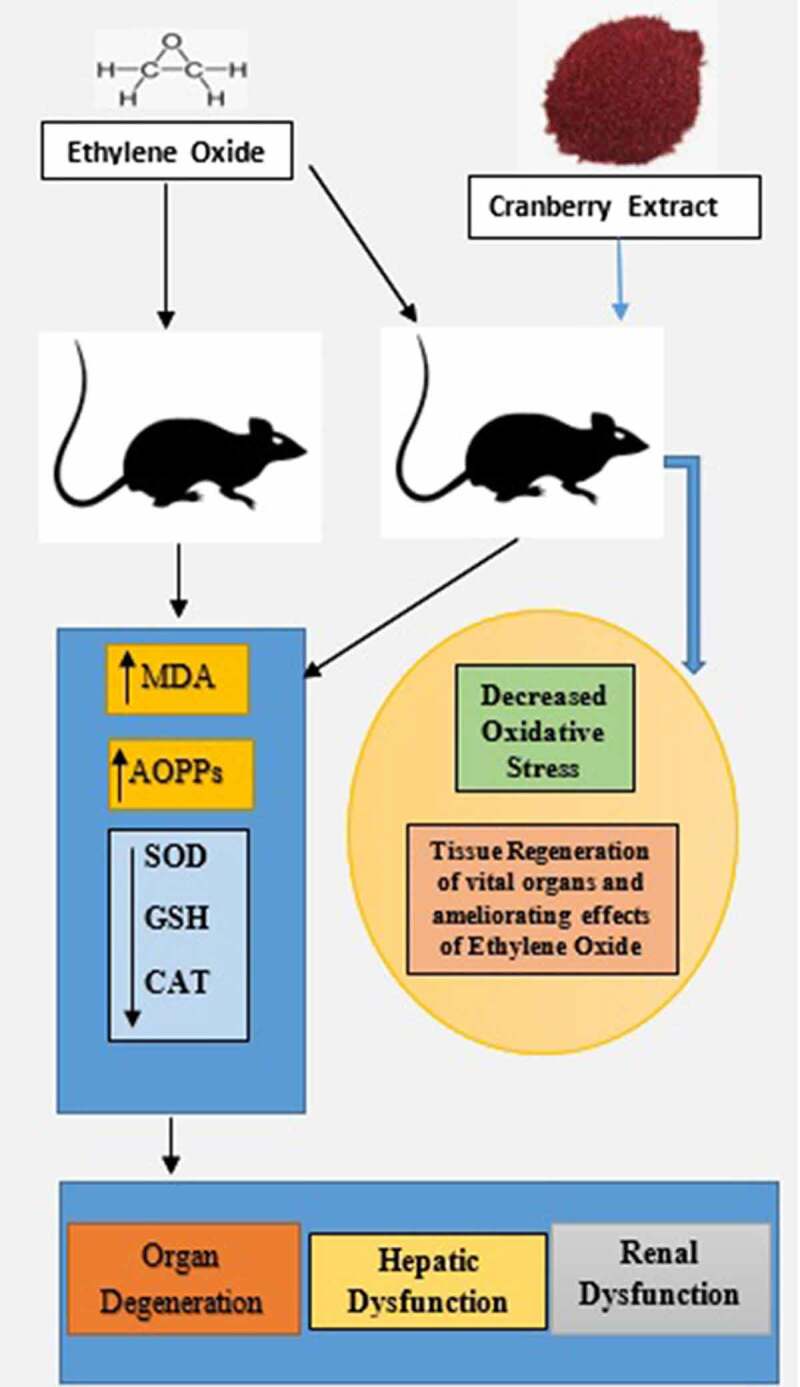

## Introduction

American cranberry (*Vaccinium macrocarpon)* is one of the major cultivated fruit crops that are native to North America. Cranberries produce a host of important secondary polyphenolic compounds, some of which are beneficial to human health. The extract of this plant has primarily been cultivated for use in industry as a preservative for storing meat. In the medical industry, its primary application is for the dressing of wounds; it is also widely used to treat oral caries and possibly possesses great anticancer properties [1]. Many fungal pathogen varieties are also susceptible to action by cultivated cranberry [2].

Ethylene oxide (EtO) is a colorless gas with a sweet odor that boils at 10.5°C (range of 10.4–10.7°C) at 764 mm Hg and melts at −111.3°C (CEFIC, 2013). Ethylene is the most widely used petrochemical in the world and is metabolized into EtO by mammalian cells [3]. EtO is ranked among the 25 chemicals with the highest production in the United States [4]. The major use of EtO (99%) is in the production of several industrial chemicals, and the remainder is used in gaseous form as a sterilizing agent, insecticide, disinfectant or fumigant and for vermin control. Fumigant or sterilizing agents are involved in a variety of facilities and materials, including medical and dental clinics and hospital equipment [5]. As a common practice, ethylene is used to control the ripening of fruits, but when it enters a living human, it can induce carcinogenic effects [6,7]. As stated globally, the increased use of these petrochemicals, as described in the previous literature, is directly responsible for increased oxidative stress and related disorders and serves as a strong related factor in the aggravation of disease [8]; therefore, offsetting the role of EtO through the use of proper antioxidants, i.e., cranberry extract, can significantly help in reducing its dangerous consequences.

Oxidative stress is the increased production and defective processing of reactive oxygen species (ROS); an excess of these species has catastrophic pro-cancerous effects, such as altering chromosomal integrity and causing genomic instability and cell proliferation [9]. ROS mediate the stimulation of activator protein-I (AP-I) and signal transduction processes related to nuclear factor kappa (NF-kB) [10]. Ethylene glycol is produced upon EtO hydrolysis, also resulting in oxidative stress. The GSH [11], SOD and CAT levels decrease, while the MDA levels significantly increase [12]. In cancerous cells, increased ROS production depletes the capacity of SOD and other antioxidative mechanisms [13]. Vigorous ROS generation causes DNA mutation, a critical step in oncogenesis that results in DNA adducts such as 8-OH deoxyguanosine [14,15]. EtO is known to cause hematopoietic cancers (leukemia, lymphosarcoma, peritoneal mesothelioma, reticulosarcoma, non-Hodgkin’s lymphoma and Hodgkin’s lymphoma), digestive cancers (papilloma, squamous cell carcinoma of the forestomach and pyloric adenocarcinoma), respiratory cancers (laryngeal carcinoma, small-cell carcinoma), urinary cancers (renal cell carcinoma, urothelial carcinoma), subcutaneous tumors, tumors of the mammary gland, uterine tumors, bone cancers, and nervous system tumors such as gliomas [16].

Cranberries contain various phytonutrients with different active constituents. Phenolic acids are important phytonutrients and they include vanillic and ferulic acids as active constituents with antioxidant and antiaging properties. Anthocyanin has cyanidins as active constituents, which block an enzyme (ornithine decarboxylase) responsible for cancer growth. Angiogenesis is also inhibited and is important for cancer proliferation [17]. The proanthocyanidins include epicatechins, which induce cell death, particularly in breast, brain, colon, ovarian and esophageal cancers [18]. Flavonoids are important phytonutrients, including the active molecule quercetin, which acts as a potent antioxidant and inhibits the growth of human breast cancer, colon adenocarcinoma and myelogenous leukemia [19]. Ursolic acid is a triterpenoid that acts as a body recomposition agent and can increase muscle mass and decrease body fat. In short, cranberries scavenge ROS and decrease the risk of cancer and other diseases. However, we lack sufficient evidence regarding the application of *Vaccinium* plant species, even though some aspects of their effectiveness are known in the medical field.

he present study attempts to explore the role of EtO-mediated increases in oxidative stress in a rat model, and then the rats were subjected to the cranberry application as the treatment option. Lastly, histopathological changes in the rats were observed to determine the toxic effects of EtO on the vital organs and the ameliorating effects of cranberry when they were administered in the rat models.

## Materials and methods

### Collection of plant materials

Fresh parts of the medicinal plant *Vaccinium macrocarpon* were dried until all their water molecules evaporated and the plants were dry enough to grind into a fine powder with a mechanical blender, and it was then transferred into airtight containers for future use.

## Preparation of plant extracts

### Solvent extraction

The crude plant extract was prepared by Soxhlet extraction method. Approximately 20 gm of powdered plant material was uniformly packed into a thimble and extracted with 250 ml of five different solvents: methanol, ethanol, ethyl acetate, chloroform and petroleum ether. The extraction process was continued for 24 hours or until the solvent in the siphon tube of an extractor became colorless. After that, the extract was placed in a beaker, kept on a hot plate and heated at 30–40°C until all the solvent had evaporated. The dried extract was kept in a refrigerator at 4°C for future use in phytochemical analysis.

## Test for phenols and tannins

The crude extract was mixed with 2 ml of a 2% solution of ferric chloride (FeCl_3_). A blue-green or black coloration indicated the presence of phenols and tannins.

## Test for flavonoids (Shinoda test)

The crude extract was mixed with a few fragments of magnesium ribbon, and concentrated hydrochloric acid (HCl) was added dropwise. Pink scarlet color appeared after a few minutes, which indicated the presence of flavonoids.

## Test for terpenoids

The crude extract was dissolved in 2 ml of chloroform and evaporated to dryness. To this end, 2 ml of concentrated sulfuric acid (H_2_SO_4_) was added and the mixture was heated for approximately 2 minutes. A grayish color indicated the presence of terpenoids

## Test for alkaloids

The crude cranberry extract was mixed with 2 ml of 1% HCl, subjected to higher temperatures and gently mixed. Mayer and Wagner’s reagents were added to the mixture. The turbidity of the resulting precipitates was viewed as evidence for the presence of alkaloids.

## Animals, diets and induction of EtO toxicity

Albino rats of the Wistar strain (100–200 g) were fed a lab-prepared diet, which was available *ad libitum*, and the rats were kept under 12 h day and night conditions. Laboratory-acclimatized rats were divided into four groups (six rats in each group) of similar average body weights (B.wt.). The animals in group (A) served as a positive control, i.e., they were healthy rats and were on a regular diet. The animals in groups B, C and D received EtO at 10 mg/ml [20]. Later, no treatment was provided to Group B, whereas Group (C) received cranberry extract at 200 mg/kg B.wt. and Group (D) received cranberry extract at 400 mg/kg B.wt. The study was approved by the Institutional Review Board and research ethical committee of the Institute of Molecular Biology and Biotechnology (IMBB) at the University of Lahore, and all the guidelines for handling animals were followed according to internationally established principles.

## Blood collection, organ separation and preservation

At the end of the experiment, blood (5 ml) was withdrawn from the veins of the rats. The serum was separated by centrifugation for 10 min at 3000 rpm and stored at −4°C until biochemical analysis. The tissues of different organs were stored in 10% formalin for histopathological examination, while the remaining tissues were used to prepare the tissue homogenate.

## Biochemical assays

### Estimation of catalase (CAT) in serum samples

Catalase was estimated using the method of Aebi (1974) [21]. In brief, using a spectrophotometer, the catalase absorbance was measured at 230 nm. The homogenized sample in pH 7.0 phosphate buffer (PB) was centrifuged at 5000 rpm. In the cuvette, 0.01 M PB, hydrogen peroxide (H_2_O_2_; 2 mM) and the sample were combined, and the reaction was started. The specific activity of catalase is reported in units/gram of sample. The absorbance was calculated with the help of a standard curve generated from known amounts of catalase.

## Estimation of thiobarbituric acid-reactive substances in serum samples

The lipid peroxidation in the samples was estimated calorimetrically using the method by Ohkawa (1979) [22] In brief, the sample (200 μl) was transferred to a test tube. Subsequently, 200 μl of 8.1% SDS, 1.5 ml of acetic acid (20%) and 1.5 ml of TBA (0.8%) were also added to the test tube, and the contents were heated for 60 min. After sample cooling, n-butanol (4 ml) was added, followed by centrifugation for 10 min at 3000 rpm. The upper organic layer was removed, and the absorbance was measured at 532 nm against a blank.

## Estimation of superoxide dismutase (SOD) in serum samples

Superoxide dismutase (SOD) was determined using the method of Kakkar (1984) [23]. In brief, the sample (100 μl) was transferred into a Falcon tube. Subsequently, sodium phosphate buffer (1.2 ml; pH 8.3; 0.052 M), phenazine methosulfate (0.1 ml; 186 μM), nitro blue tetrazolium (0.3 ml; 300 μM) and NADH (0.2 ml; 750 μM) were also added, and the reaction was started. Following incubation for 90 seconds at 30°C, glacial acetic acid (0.1 ml) was added to stop the reaction. Then, 4.0 ml of n-butanol was added to the Falcon tubes, followed by centrifugation for 10 min at 4000 rpm. The upper layer (butanol) was removed, and the absorbance was measured at 560 nm.

## Estimation of reduced glutathione (GSH) in serum samples

Serum and liver glutathione (GSH) were estimated using the method by Moron (1979) [24]. A chromophore, TNB (trinitrobenzene) (absorbance at 412 nm), and oxidized glutathione (GSSG) are produced when GSH reacts with Ellman’s reagent (DTNB). The calculated GSH level is the sum of the levels of the oxidized (GSSG) and reduced (GSH) forms of glutathione.

## Determination of AOPPs

The AOPP levels were estimated according to the method described by Witko-Sarsat et al. (1996) [25]. Plasma was diluted with 1:5 PBS and chloramine T for calibration. Next, 10 µl of 1.16 M potassium iodide and 20 µl of acetic acid were added to each sample, and the absorbance was measured at 340 nm against the blank.

## Histopathological studies of different organs

Histopathological changes in the tissues of different organs stored in formalin were observed microscopically after hematoxylin and eosin staining.

## Statistical analysis

Statistical analyses were performed by two-way analysis of variance (ANOVA) followed by Duncan’s multiple range test (DMR test) with SPSS v.21 and Co-stat. The values are reported as the mean ± SD, and P values < 0.05 were considered statistically significant.

## Results

*Vaccinium macrocarpon* was initially extracted using different solvents, namely methanol, ethanol, chloroform, ethyl acetate and petroleum ether. The yields are reported as mg/100 g of crude extract. Petroleum ether yielded the highest extract concentration, followed by ethyl acetate, ethanol, methanol and chloroform (as shown in Table 1). Thus, the petroleum ether extract was selected for phytochemical analysis. Table 2 shows the list of phytochemicals that were reported in the plant extract, and according to the reported findings, the most common and extensively reported phytochemicals were flavonoids, followed by triterpenoids, phenols, carotenoids, alkaloids, tannins and anthocyanins.

**Table 1.** Percent yield when different solvents were used (mg/100 g crude extract)

Medicinal plant	Methanol	Ethanol	Chloroform	Ethyl acetate	Petroleum ether
***Vaccinium macrocarpon***	3.98 ± 0.87	5.76 ± 0.35	3.76 ± 0.11	6.56 ± 1.76	13.76 ± 2.65

Values are in mg/100 g crude extract

**Table 2.** Total phenolic, tannin, alkaloid and flavonoid contents in the petroleum ether *Vaccinium macrocarpon* extract

Medicinal plant	Phenols	Tannins	Alkaloids	Flavonoids	Carotenoids	Triterpenoids	Anthocyanin
***Vaccinium macrocarpon***	27.87 ± 2.54	11.08 ± 1.76	17.87 ± 2.76	56.87 ± 3.98	24.09 ± 3.87	34.76 ± 2.98	10.78 ± 0.67

Represented values are the average of three analyses (mean) ± standard deviation (SD) in mg of GAE/g of extract, where GAE is gallic acid equivalents.

Table 3 lists the levels of oxidative stress markers reported among the four groups of rats after the statistical analysis, and it shows that the levels of these markers differed significantly in the group of rats that were treated. The MDA and AOPPs levels were reported to be significantly higher in Group B (8.12 ± 1.24 nmol/ml and 9.25 ± 1.23 pg/ml) than in Group A (1.32 ± 0.25 nmol/ml and 3.25 ± 0.05 pg/ml), for signifying alleviated oxidative damage among the rats treated with EtO. However, the MDA and AOPP levels remained (5.62 ± 1.25 nmol/ml, 4.12 ± 0.95 pg/ml and 2.22 ± 0.57 nmol/ml, 5.93 ± 1.02 pg/ml) in Groups C and D, respectively, which reports the ameliorating effects of cranberry extract in the rats receiving EtO. Similarly, the SOD, GSH and CAT levels were significantly lower (39.26 ± 4.26 nmol/ml, 1.02 ± 0.01 nmol/ml and 13.23 ± 1.26 nmol/ml) in the EtO-treated group labeled group B, which signifies an altered antioxidative response in the rats treated with EtO compared to Group A (84.26 ± 12.25 nmol/ml, 8.96 ± 1.11 nmol/ml and 30.26 ± 1.99 nmol/ml, respectively). However, treating the rats with the cranberry extract at doses of 200 mg/kg and 400 mg/kg B.wt as in Groups C and D led to significantly improved levels of antioxidants that showed the significance of the extract in treating EtO-induced oxidative stress among the groups of rats, i.e., the levels of SOD, GSH and CAT remained (53.26 ± 7.25 nmol/ml, 6.23 ± 1.24 nmol/ml, 25.36 ± 3.25 nmol/ml and 75.26 ± 8.32 nmol/ml, 7.26 ± 2.25 nmol/ml, 25.36 ± 3.25 nmol/ml) in Groups C and D, respectively. Active ingredients of the cranberry extract are shown in Table 4.

**Table 3.** Antioxidative profile of different variables in EtO-induced, toxicity-treated rats receiving an extract of *Vaccinium macrocarpon.*

Variables	Control	EtO (10 mg/ml B.wt)	EtO (10 mg/ml B.wt + Cranberry 200 mg/kg B.wt)	EtO (10 mg/ml B.wt + Cranberry 400 mg/kg B.wt)	p-value
**AOPPs**	3.25 ± 0.05	9.25 ± 1.23	4.12 ± 0.95	5.93 ± 1.02	0.042
**GSH**	8.96 ± 1.11	1.02 ± 0.01	6.23 ± 1.24	7.26 ± 2.25	0.039
**SOD**	84.26 ± 12.25	39.26 ± 4.26	53.26 ± 7.25	75.26 ± 8.32	0.036
**MDA**	1.32 ± 0.25	8.12 ± 1.24	5.62 ± 1.25	2.22 ± 0.57	0.035
**CAT**	30.26 ± 1.99	13.23 ± 1.26	27.26 ± 2.35	25.36 ± 3.25	0.037

**Table 4.** Active constituents of the plant extract

Phytonutrients	Active Constituents
Phenolic acids	Hydroxybenzoic acid (including vanillic, hydroxycinnamic, caffeic and ferulic acids)
Proanthocyanidins	Epicatechins
Anthocyanin	Cyanidins
Flavonoids	Quercetin
Triterpenoids	Ursolic acid

## Histopathological study

### Histopathology findings in rat hearts

Photomicrographs of EtO-treated hearts indicated severe cardiac myocyte damage, with the loss of myocardial fibers and vacuolated cells. Additionally, the normal branching of cardiac muscle cells was disrupted, with faded nuclei and a severe shrinkage of the cells. The degree of cell damage following exposure to EtO stress is dependent on the rate of ROS formation and on the efficiency and capacity of EtO and repair mechanisms. Studies (Aleksandra et al., 2013) have revealed that myocardial injury occurs due to occupational or environmental exposure to toxins such as EtO. Histopathological studies (Aleksandra et al., 2013) have revealed that particulate matter leads to marked injury and inflammatory changes in the myocardial tissue of experimental rats. However, treating with cranberry at 400 mg/kg resulted in near normalcy by ameliorating the changes, indicating that the treatment was useful (Figure 2).

**Figure 1.**
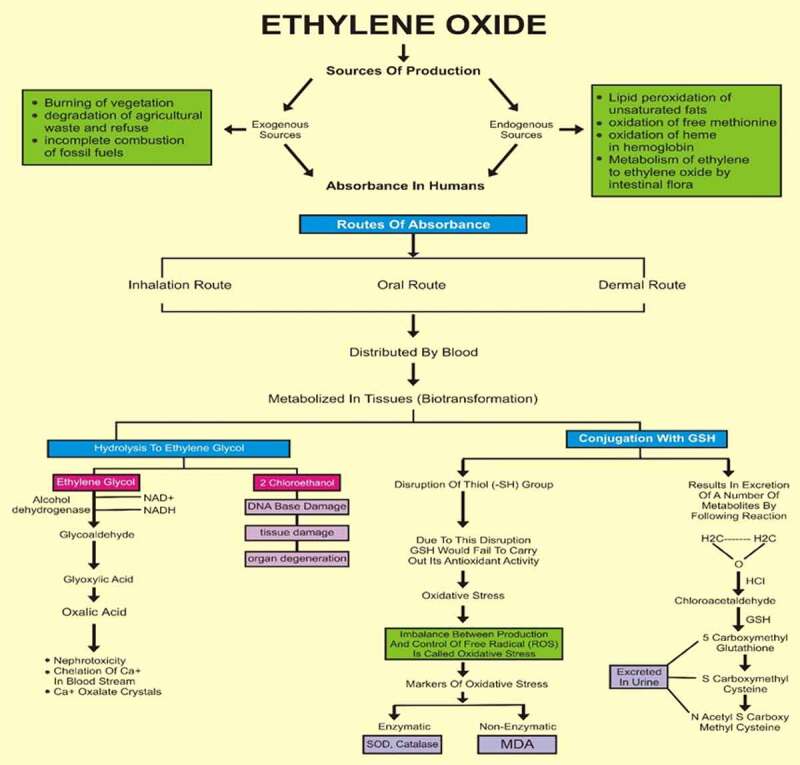
Ethylene oxide, an epoxide, has basically two sources, i.e., exogenous and endogenous. The burning of vegetation, the degradation of agricultural waste and refuse, and the incomplete combustion of fossil fuels are exogenous sources. Endogenous sources include the lipid peroxidation of unsaturated fats, the metabolism of ethylene to ethylene oxide by intestinal flora, the oxidation of heme in hemoglobin and free methionine. Whatever the source of production, the result of the encounter is absorption by the body, which can occur by three routes: inhalation, oral or dermal. After being taken up by any of these routes, ethylene oxide is widely distributed by the blood throughout the whole body. It is metabolized in tissues by two major pathways. One pathway is conjugation with GSH, resulting in the production of metabolites by a series of reactions, the end products of which are excreted in urine. During conjugation, the disruption of thiol (-SH) groups leads to the impaired function of GSH, that results in oxidative damage. The markers of oxidative stress that we measured were SOD, catalase, MDA and AOPP. The first two are enzymatic, while the latter two are non-enzymatic. The other pathway involves hydrolysis to form ethylene glycol, which later forms products such as oxalic acid, which induce nephrotoxicity, the chelation of Ca^2+^ in the bloodstream and the formation of Ca^2+^-oxalate crystals in the kidneys. In addition to ethylene glycol, 2-chloroethanol is formed, which causes DNA damage, tissue damage and organ degeneration

**Figure 2.**
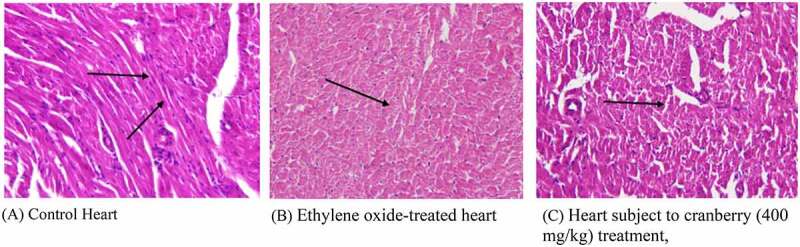
Histopathological examination of the hearts of rats before and after treating with cranberry (scale bar 35 microns with 40x magnification), indicating severe cardiac myocyte damage with a loss of myocardial fibers and vacuolated cells

## Histopathology of rat kidneys

**Figure 3.**
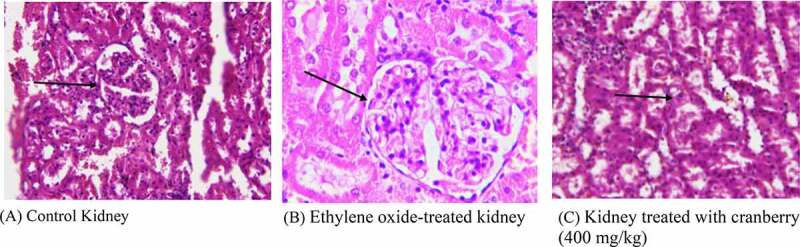
Histopathological examination of the kidneys of rats before and after treatment with cranberry (scale bar 35 microns with 40x magnification). Although the architecture of the kidney tissue remained normal, EtO dilation of renal tubules could be observed

The kidney tissue of the control rats had a normal architecture. However, the photomicrograph of EtO-treated kidneys showed the marked toxicity of EtO; there was dilation of the renal tubules as well as degeneration of the epithelial cells lining the renal tubules. Nevertheless, the glomeruli were spared and relatively less affected. The tissue of animals that had received cranberry treatment at both doses had normal architecture, as observed in the control rats (Figure 3).

## Histopathology of rat liver

**Figure 4.**
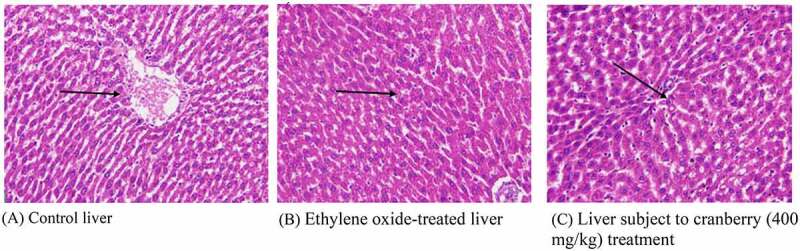
Histopathological examination of the livers of rats before and after treatment with cranberry (scale bar 35 microns with 40x magnification). Following exposure to EtO, the normal structural organization of the hepatic lobules indicated damaged cells with a loss of characteristic cord-like arrangements

Normally, the liver is divided into three zones based on oxygen supply. Zone 1 encircles the portal tracts through which the oxygenated blood from the hepatic arteries enters. Zone 3 is located around the central veins around which oxygenated blood is poor. Zone 2 is in between Zones 1 and 3. A histopathological examination of the liver tissue of the control rats indicated a normal architecture. Following treatment with EtO, the normal structural organization of the hepatic lobules indicated damaged cells with a loss of characteristic cord-like arrangements. The central and portal veins were clogged. Significant numbers of hepatic cells were impaired, and their physical appearance indicated that some cells had undergone marked cytoplasmic vacuolization. Wide degeneration of the hepatocytes was also clearly visible. Cytoplasmic feathering in the liver transport system, demonstrating clear disturbance, also confirmed the effects of the chemical. Cranberry treatment nevertheless ameliorated most of the damage observed in the liver architecture (Figure 4).

## Histopathology of rat lungs

**Figure 5.**
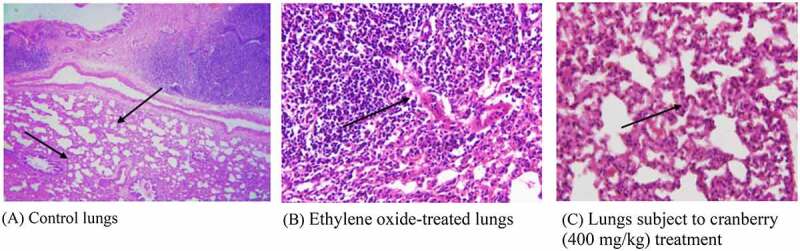
Histopathological examination of the lungs of rats before and after treatment with cranberry (scale bar 35 microns with 40x magnification). Changes can be observed in the architecture of connective tissues, smooth tissues and bronchioles in EtO-treated lung tissues

Photomicrographs of EtO-treated lungs indicated permeation by chronic inflammatory cells, leading to the broadening of the alveolar septa. However, there were changes in the architecture of the connective tissues, smooth tissues and bronchioles in EtO-treated lung tissues, indicating the presence of inflammation and pulmonary fibrosis. The thickening of the alveolar septa could be attributed to inflammation, blemishing or the presence of extra fluid (edema). There was a visible narrowing of the cells and a decrease in the cytoplasmic content. Cranberry-treated tissue, however, had a normal architecture comparable to that of the control (Figure 5).

## Histopathology of rat stomachs

**Figure 6.**
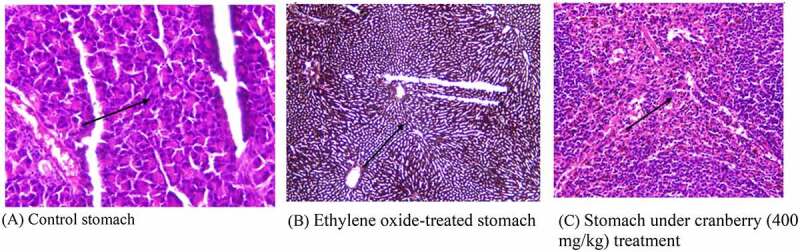
Histopathological examination of the stomach of rats before and after treating with cranberry (scale bar 35 microns with 40x magnification). The photomicrograph of the EtO-treated stomach clearly shows a decrease in the cytoplasmic content and a narrowing of the cells

The mucosa of the control rats in the stomach, which is covered by simple columnar epithelium, had a normal architecture. The mucosa and submucosa were intact, with no edema or infiltration of the cells. The photomicrograph of the EtO-treated stomach clearly shows a decrease in the cytoplasmic content and a narrowing of the cells, with fewer nuclei observed, as well as a disturbed vascular system, which was confirmed by the changes in the color of the cells. Additionally, the blood flow was arrested. There was mucosal and submucosal edema, infiltration of inflammatory cells, and hemorrhaging of the mucosa. Treating with cranberry again reversed the changes to a near-normal state (Figure 6).

## Histopathology of rat testis

**Figure 7.**
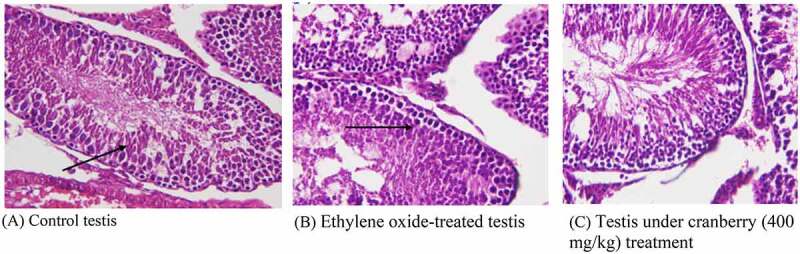
Histopathological examination of the testes of rats before and after treatment with cranberry (scale bar 35 microns with 40x magnification). Photomicrographs of EtO-treated testes indicated edema leading to the separation of the cell lining of the seminiferous tubules, the seizure of spermatogenesis, and focal necrosis

Photomicrographs of EtO-treated testes indicated marked histological changes. Edema led to the separation of the cell lining of the seminiferous tubules, seizures in spermatogenesis, and focal necrosis. The transport systems of the cells were badly disturbed, and there was clear cellular degeneration. The nuclei were faded, with decreased spermatogenesis. There was also decreased spermatogonia and disrupted sperm flagella. Tissues that had received cranberry treatment again had normal architecture (Figure 7).

## Discussion

The current research extensively confirmed the protective effects of *V. macrocarpon* against oxidative stress and related toxic effects. The given activity of the extract was primarily attributed to the phytoconstituents present in the extract. The phytochemical analysis of the plant in the different media yielded a number of flavonoids, carotenoids, triterpenoids and phenols. The highest yield of the constituents was reported in petroleum ether as the solvent. The literature reports that the present constituents are significantly involved in a number of antiproliferative and anticancer activities, i.e., flavonoids are reported to inhibit the proliferative activity of tumor cell lines and induce apoptosis. Quercetin is an extensively studied dietary flavonoid and a potent antioxidant with a number of active components that contribute to the antiproliferative activity of the extract. These proteins are involved in inhibiting the function of proteins that are involved in signal transduction and gene transcription [26]. Likewise, the second-most frequently reported phytochemicals are triterpenoids, which also exhibit antiproliferative and antitumor activities. Triterpenoids can also limit the incidence of vascular diseases, i.e., neurodegenerative disorders related to age and atherosclerosis [27,28]. Similarly, phenols and carotenoids have significant protective activities, i.e., in the case of urinary tract infections [29]. Carotenoids have also been reported to have high antioxidant activities with other phytoconstituents, such as tannins, alkaloids and anthocyanins, which are effective even in trace amounts. According to Blumberg et al. (2013), the amount of anthocyanins is remarkably high in cranberries [30]. Due to their role in the absorption of light energy in photosynthesis, carotenoids are an essential component of all plants, and thus, plants are believed to play significant roles as antibacterial and antifungal agents. Dried cranberry powder was used in the current study, because the literature reports that it has higher phytoconstituent levels than liquid cranberry powder [31].

The findings of this study help to support the finding that rats exposed to EtO were reported to be more susceptible to developing related toxic effects, including increased oxidative stress and damage to vital organs. Administering cranberry extract to these rats has significantly ameliorating effects against ethylene-induced toxic effects. Moreover, the histopathological results from different organs also confirmed the antitumorigenic properties of *Vaccinium macrocarpon* in rats exposed to EtO. EtO in rats was distributed through the blood and primarily metabolized in tissues, resulting in hydrolysis and conjugation. Following hydrolysis, secondary products such as ethylene glycol and 2-chloroethanol were produced. Ethylene glycol is converted into glycolaldehyde, ultimately forming oxalic acid and resulting in tissue injury. Notably, 2-chloroethanol causes organ degeneration by DNA damage. Ethylene glycol forms conjugates with glutathione (GSH), resulting not only in its excretion through urine in the form of S-carboxymethyl GSH and S-carboxymethyl cysteine but also in its disruption of thiol (-SH) groups, as shown in Figure 1. Both effects lead to decreases in the GSH levels, resulting in oxidative stress [11]. Our results depicted a reduction in GSH levels in the toxic ethylene group. There was also a reduction in antioxidant enzymes, i.e., SOD and catalase (CAT), and thus a decrease in the processing of oxidants, resulting in the production of superoxide anions (O_2_¯), which mediate stimulation activator protein-I (AP-I) and signal transduction processes involved with nuclear factor kappa (NF-kB) [10], leading to cancer. We also noted reduced levels of these antioxidants. By contrast, the AOPP and MDA levels increased, because O2^. –^ causes protein carbonylation/oxidation and lipid peroxidation. Moreover, the plasma levels of antioxidants, i.e., SOD, CAT and GSH, were significantly improved in response to treatment with cranberry extract [32,33]. The findings of the study were also supported by the literature, which showed that the levels of AOPPs and MDA were significantly decreased after the administration of cranberry extract [34].

Similarly, significant effects were observed in the histopathological studies on rats receiving cranberry extracts followed by ethylene-induced toxicity, because the findings could be observed at the plasma level. EtO-induced histopathological changes were examined in various tissues of rat models. In the heart, myocardial fiber loss, vacuolated cells, disruption of cardiac myocytes and branching and fading of the nuclei were observed. The narrowing of cells with a decreased cytoplasmic content and a disturbed vascular system was evidenced by the color of cells in the stomach. The loss of characteristic cord-like structures, such as the arrangement of hepatocytes, marked cytoplasmic vacuolization, fatty degeneration and clogging of central and portal veins, occurred in the liver. Damage to renal tubular epithelial cells, tubular dilation and less affected glomeruli were observed in the kidneys. The thickening of the alveolar septa, cell narrowing with reduced cytoplasmic content, and inflammatory and fibrotic changes were observed in the lungs. In the testis, the separation of the cell lining of seminiferous tubules, fewer spermatogonia and the disruption of sperm flagella were noted. When cranberry was administered at 400 mg/kg B.wt, these histopathological changes reverted to an almost normal state. To the best of our knowledge, we are the first to report the use of EtO to generate oxidative damage and histopathological changes along with their reversion upon treatment with cranberry. The histopathological findings of the current study also indicated the ameliorating effects of the plant extract in the tissues of several vital organs in rats. As shown, exposure to EtO indicated severe cardiac myocyte damage with a loss of myocardial fibers and vacuolated cells, dilation of renal tubules, damaged hepatic cells with a loss of characteristic cord-like arrangements, changes in the architecture of connective tissues, smooth tissues and bronchioles in EtO-treated lungs; in the stomach, EtO was responsible for a decrease in the cytoplasmic content and a narrowing of the cells, and finally, in the testis, EtO led to edema and the separation of the cell lining of seminiferous tubules, the seizure of spermatogenesis, and focal necrosis. However, histopathological findings in rats treated with plant extract showed significant repair and cranberry treatments were responsible for significant repair in these tissues in rats exposed to EtO.

## Conclusion

The present study concluded that major toxic effects were observed in rats following EtO exposure, as witnessed from various oxidative stress markers and the histopathological degeneration of the studied organs. Significantly lower antioxidant levels were observed during EtO intoxication, resulting in the development of tumorigenesis in various tissues. Administering petroleum ether cranberry extract to rats showed promising results in the inhibition of the tumorigenic effects induced by EtO along with the ameliorating effects of cranberry on ethylene-induced toxicity. In the future, more research on larger groups of rats and *in vivo* models is recommended to clarify the medicinal implications of using cranberry extracts.

## Highlights

- The organ-healing effect of cranberry was studied in *Rattus norvegicus*.
- Major antioxidant recovery was observed in cranberry-treated (400 mg/kg) rats.
- A sharp improvement in stress-related markers was also observed after cranberry treatment.
